# Global parameter estimation methods for stochastic biochemical systems

**DOI:** 10.1186/1471-2105-11-414

**Published:** 2010-08-06

**Authors:** Suresh Kumar Poovathingal, Rudiyanto Gunawan

**Affiliations:** 1 Department of Chemical and Biomolecular Engineering, National University of Singapore, 4 Engineering Drive 4, 117576, Singapore

## Abstract

**Background:**

The importance of stochasticity in cellular processes having low number of molecules has resulted in the development of stochastic models such as chemical master equation. As in other modelling frameworks, the accompanying rate constants are important for the end-applications like analyzing system properties (e.g. robustness) or predicting the effects of genetic perturbations. Prior knowledge of kinetic constants is usually limited and the model identification routine typically includes parameter estimation from experimental data. Although the subject of parameter estimation is well-established for deterministic models, it is not yet routine for the chemical master equation. In addition, recent advances in measurement technology have made the quantification of genetic substrates possible to single molecular levels. Thus, the purpose of this work is to develop practical and effective methods for estimating kinetic model parameters in the chemical master equation and other stochastic models from single cell and cell population experimental data.

**Results:**

Three parameter estimation methods are proposed based on the maximum likelihood and density function distance, including probability and cumulative density functions. Since stochastic models such as chemical master equations are typically solved using a Monte Carlo approach in which only a finite number of Monte Carlo realizations are computationally practical, specific considerations are given to account for the effect of finite sampling in the histogram binning of the state density functions. Applications to three practical case studies showed that while maximum likelihood method can effectively handle low replicate measurements, the density function distance methods, particularly the cumulative density function distance estimation, are more robust in estimating the parameters with consistently higher accuracy, even for systems showing multimodality.

**Conclusions:**

The parameter estimation methodologies described in this work have provided an effective and practical approach in the estimation of kinetic parameters of stochastic systems from either sparse or dense cell population data. Nevertheless, similar to kinetic parameter estimation in other modelling frameworks, not all parameters can be estimated accurately, which is a common problem arising from the lack of complete parameter identifiability from the available data.

## Background

Mathematical models form a cornerstone of systems biology and these models are usually constructed from available biological knowledge and data, which once validated, are subsequently analyzed to address specific biological questions. Many canonical modelling frameworks, from statistical Bayesian networks to differential equations, have been applied to capture a wide-variety of biological behaviours. Specifically, the dynamics related to cellular processes that involve low copy number of molecules, such as mRNA transcription, are best described as random and noisy events [1]. For example, cells in an iso-genetic population do not necessarily assume the same biological state, but rather exhibit variegated genetic expressions [2,3]. In these examples, the distribution of cells is simulated by stochastic models that describe the probability density function (PDF) of cellular states. However, unlike differential equation models, the identification of stochastic models from experimental data of single cell or cell population data are not yet routine.

Despite the availability of high-throughput cell biology, the estimation of unknown (kinetic) model parameters from experimental data is still considered as the bottleneck in biological model identification, especially for dynamical models [4,5]. The difficulty is generally attributed to the informativeness of the data, or the lack thereof, a property that is proportional to not only the quantity, but also the quality of data. Furthermore, in dynamical models, the time resolution of data is naturally of great importance. In recent years, advances in bio-imaging allow for real time measurements of cellular components such as mRNAs and proteins in individual cells through the use of fluorescent proteins [2,3,6-8]. Such measurements provide more in-depth and informative data about the states of a cell and variability in a cell population, than the traditional lumped measurements from cell culture lysate or tissue homogenate. The purpose of this work is to develop practical methods that can efficiently use these data in the parameter estimation framework for stochastic biochemical systems.

Chemical master equation (CME) is the most commonly adopted modelling framework to describe stochastic cellular dynamics [1-3] and thus is used as a benchmark application in this work. The estimation of unknown kinetic parameters from data in CME and other stochastic models has not been adequately addressed in the literature. Many of the published CME models use rate constants that are scaled from deterministic parameter values or selected ad-hoc to replicate desired behaviour. Since the low-copy-number random events can generate dynamics that are characteristically different from those in thermodynamic or deterministic limit [9,10], deterministic model parameters identified from data collected under this limit or averaged over cell populations can be misleading. Furthermore, fitting deterministic models (e.g. ordinary differential equation) to stochastic data has been shown to give poor parameter estimates and model prediction [11]. Among the existing parameter estimation methods for stochastic biological models, some rely on Bayesian inference based on the stochastic differential equation [12,13], while others are based on maximum likelihood (ML) methods. One ML method obtains parameter estimates by fitting transition density functions of stochastic differential equations in biochemical pathways [11]. A similar approach based on the ML of transitional probabilities requires measurements of the state trajectories at very fast sampling rate, whereby reactions are assumed to occur at most twice in a sampling time interval [14]. The fast sampling requirement makes this approach impractical, since biological data are typically sparse.

In this work, three kinetic parameter estimation methods for stochastic models were developed based on two criteria: maximum likelihood (ML) and density function distance (DFD). Two scenarios of practical application were considered involving both sparsely and densely populated datasets (i.e. low and high replicates). Since the distribution density functions are commonly constructed using histograms, an important aspect related to the binning strategy and the noise associated with finite sampling, has been incorporated in the parameter estimation framework. The efficacy of each method was evaluated and compared based on applications to three CME case studies: RNA dynamics in *Escherichia coli*, gene expression network of galactose uptake model in *Saccharomyces cerevisiae*, and a bimodal system comprising of a genetic toggle switch in *E. coli*. Despite the use of CME models here, the methods are generally applicable to other stochastic models in which the system behaviour or output can be characterized by a PDF of the states.

## Methods

### Chemical Master Equation

Consider a well mixed volume Ω containing *N *species participating in *M *biochemical reactions. The CME of this system is given by [15]:

(1) $$\begin{array}{l} \frac{dP\left(x,t|x_{0},t_{0}\right)}{dt}=\sum_{j=1}^{M}a_{j}(x-\nu_{j},k)P(x-\nu_{j},t|x_{0},t_{0})\\ \;-a_{j}(x,k)P(x,t|x_{0},t_{0}), \end{array}$$

where the state **x **is an *N*-dimensional vector indicating the number of molecules of each species in the volume Ω, the density function *P*(**x**, *t*|**x**_0_, *t*_0_) denotes the probability that the system assumes the state configuration **x**_*j *_at time *t*, given the initial condition **x**_0 _at time *t*_0_, the vector **ν**_*j *_gives the stoichiometric change in the molecular count of each species due to a single *j*-th reaction event, and **k **is the kinetic parameter vector. The function *a*_*j*_(**x**, **k**) is known as the propensity function, where *a*_*j*_(**x**, **k**)d*t *gives the probability of the *j*-th reaction to occur in the time interval *t *and *t+*d*t *given the state **x **and parameters **k**. Due to the curse of dimensionality with increasing number of reacting species, the analytical solution of a CME is usually difficult, if not practically impossible, to obtain even for moderately sized systems [16].

In this work, Stochastic Simulation Algorithm (SSA) [16] was used to generate *in silico *experimental data for the purpose of parameter estimation and to solve for the PDF of the CME model. Briefly, at any given time and state configuration, the algorithm takes two uniform random numbers, from which the time to the next reaction and the reaction index are determined as a function of the propensities [16,17]. The histogram should reflect the true state PDF in the limit of the number of realizations tending to infinity. Since only a finite number of data samples are computationally feasible and experimentally practical, the error associated with histogram binning strategy is important, but this is not often discussed in existing literature of the CME. The shape of the resulting density function is known to be sensitive to the number and size of the bins, and the optimal binning distribution need not be of uniform sizes [18]. Characteristic features of a distribution such as bimodality may not be apparent when using bins that are too wide, while histograms can be significantly affected by random fluctuations associated with a small number of data points in bins that are too narrow. Although there is no hard and fast rule on the selection of bin sizes, the minimum number of realizations in each bin should typically range between 5 and 20 [19]. Unless stated otherwise, the histograms here are constructed such that each bin contains no fewer than ten occurrences. The noise due to the histogram construction using finite size random sample will be taken into account in the parameter estimation below.

In practice, the choice of numerical solvers for model equations determines the performance of any parameter estimation methods. For CME, there has been a tremendous development of numerical algorithms for computing the PDF solution, directly [20-22] or indirectly [15,16,23]. The SSA was selected in this work because this algorithm is equivalent to the CME [16,17], motivating its use to generate *in silico *data. Consequently, the CME model was also solved using SSA, such that the efficacy of the proposed methods can be evaluated independently from the solvers. In this case, deficiencies of SSA will appear equally in both *in silico *data and the model solution.

### Parameter Estimation Methods

The methods developed here are formulated as a minimization of distance measures between model predictions and experimental data. The first method makes use of the common likelihood function and the second involves a distance metric between density functions as predicted by the CME and the data. When experimental error is known or can be determined from data, this noise should be accounted for in the PDF solution. In this work, the error is assumed to be independent and identically distributed (i.i.d.) random samples from a normal distribution with zero mean and variance σ^2 ^(*N*(0,*σ*^2^)), which are then added to the SSA realizations.

#### Maximum Likelihood (ML) Method

The first estimation criterion is the likelihood function given by

(2) $$L(k)=\prod_{j=1}^{m}\prod_{i=1}^{n}f\left(o_{i}^{j},t_{i};k\right),$$

where the *j-*th experimental replicate $\left\{o_{1}^{j},o_{2}^{j},...o_{n}^{j}\right\}$ are taken at time points {*t*_1_, *t*_2_, ... *t*_*n*_} for *j *= 1, 2, ..., *m *(i.e. the experiments are done in *m *replicates). The likelihood function $f\left(o_{i}^{j},t_{i};k\right)$ is given by the CME model, which in this case is evaluated from the density function histogram of SSA realizations. The parameter estimation is then formulated as maximization of the likelihood function given by

(3) $$\begin{array}{l} k^{*}=\arg\underset{k}{\max}L(k)\\ \;=\arg\underset{k}{\max}\prod_{j=1}^{m}\prod_{i=1}^{n}f\left(o_{i}^{j},t_{i};k\right)\\ \;=\arg\underset{k}{\max}\prod_{j=1}^{m}\prod_{i=1}^{n}P\left(o_{i}^{j},t_{i}|x_{0},t_{0}\right), \end{array}$$

where *P*(**o**, *t*_*i*_|**x**_0_, *t*_0_) is the state PDF reconstructed from SSA simulations, with added Gaussian i.i.d. noise **ε **∈ *N*(0,*σ*^2^) when appropriate, i.e. the state trajectory is simulated as **o **= **x **+ **ε **rounded to the nearest integer. For brevity, from hereon *P*(**o**, *t*_*i*_|**x**_0_, *t*_0_) will be denoted by *P*(**o**, *t*_*i*_). Specific details of the accounting of experimental errors can be found in the description of the case studies in the results section. To avoid numerical underflows, the log-likelihood formulation of the objective function (3) is used in this work, giving

(4) $$\begin{array}{l} k^{*}=\arg\underset{k}{\min}-\log L(k)\\ \;=\arg\underset{k}{\min}\sum_{j=1}^{m}\sum_{i=1}^{n}-\log P\left(o_{i}^{j},t_{i}\right). \end{array}$$

#### Density Function Distance (DFD) Method

The next two estimation methods are based on the minimization of state density function distance, similar to a divergence measure between two distribution functions, such as the Kullback-Leibler distance [24]. In particular, two estimation criteria are considered using the probability density function and cumulative density function (CDF). In the PDF distance method, the objective of the parameter estimation is to minimize the difference between the PDF of the experimental data and SSA simulations, as follows

(5) $$k^{*}=\arg\underset{k}{\min}\sum_{i=1}^{n}\sum_{l=1}^{L-1}\frac{{\left(P_{e}\left(o_{l},t_{i}\right)-P\left(o_{l},t_{i}\right)\right)}^{2}}{s_{l,i}^{2}},$$

where *P*_*e*_(**o**_*l*_, *t*_*i*_) denotes the experimental PDF constructed using a histogram with *L *bins and **o**_*l *_is arbitrarily taken to be the centre of each bin. Unless stated otherwise, the binning strategy is referenced to the experimental data and the same binning distribution is used for the SSA simulations. The last bin represents an extra degree of freedom due to normalization of the sum (integral) of the PDF to 1, and thus not included in the optimization procedure. The weighting factor $s_{l,i}^{2}$ is an estimate of the variance of the *l*-th bin probability at time *t*_*i *_arising due to finite random sampling. The process of classifying *N *elements from either the experimental data or SSA realizations into bins of a histogram can be assumed as a binomial process and thereby the variance of the bin frequency is computed according to

(6) $$s_{l,i}^{2}=\frac{P_{e}\left(o_{l},t_{i}\right)\left(1-P_{e}\left(o_{l},t_{i}\right)\right)}{N}.$$

As a reliable construction of a PDF typically requires a large number of replicates, the PDF distance may not be appropriate when only few replicates of data are available. On the other hand, the ML method above can be applied to datasets with low replicates, as it does not require the construction of a density function from the experimental data.

The second criterion considers the minimization of the differences between the CDF constructed using the experimental data and the SSA realizations, given by

(7) $$k^{*}=\arg\underset{k}{\min}\sum_{i=1}^{n}\sum_{l=1}^{L-1}\frac{{\left(F_{e}\left(o_{l},t_{i}\right)-F\left(o_{l},t_{i}\right)\right)}^{2}}{S_{l,i}^{2}},$$

where the CDF *F*_*e*_(**o**_*l*_, *t*_*i*_) gives the probability to obtain an experimental observation **o **<**o**_*l*_, and *F*_*e*_(**o**_*l*_, *t*_*i*_) and *F*(**o**_*l*_, *t*_*i*_) denote the CDF constructed from the cumulative sums of the PDF, $\sum_{k=1}^{l}P_{e}\left(o_{k},t_{i}\right)$ and $\sum_{k=1}^{l}P\left(o_{k},t_{i}\right)$, respectively. Similar to the PDF criteria, the weighting factor $S_{l,i}^{2}$ is estimated using a binomial assumption to give

(8) $$S_{l,i}^{2}=\frac{F_{e}\left(o_{l},t_{i}\right)\left(1-F_{e}\left(o_{l},t_{i}\right)\right)}{N}.$$

The binning distribution can be kept the same as the PDF, but this need not be necessarily so. Unlike PDF, the shape of CDF is less sensitive to noise from finite sampling, with the exception of the tail ends of the CDF near the minimum and maximum values of the states. An alternate formulation with a finer binning strategy gives a similar performance to the objective function above (data not shown). The lesser sensitivity to noise also makes the CDF distance method applicable to sparse datasets (low replicates), in which case the binning strategy is done based on the SSA realizations.

### Global Optimization Algorithm

Aside from model solvers, the effectiveness of any parameter estimation methods also depends on the ability to find the global optima to the minimization problems. In the case of stochastic models, the error landscape is anticipated to be highly stochastic due to noise from finite experimental data points, which prevents the use of any optimization algorithms involving gradient search. Here, a variant of evolutionary algorithms, called Differential Evolution (DE), is used as a general purpose global optimization algorithm. This method can effectively handle diversified objective function planes [25], and like other evolutionary algorithms such as genetic algorithm (GA), DE starts with a random population member and looks for the global optima by generating new population members using successive recombination and mutations based on the original parent population. However, unlike GA, DE uses floating point instead of bit string encoding, and arithmetic operations instead of logical rules, thereby providing a greater flexibility in the parameter search. Among the settings of DE, the population size and total number of generations are tuned in the case studies below based on the dimensionality of the problem (i.e. number of parameters) and the choice of parameter estimation method, respectively. The remaining parameters are maintained at previously suggested values [25]. The convergence and termination of the optimization can be based on the improvement of the best objective function in the population, standard deviation of the population vector, or maximum difference between the best and worst population member. A combination of several of these criteria can provide an efficient and robust termination criterion [26]. Since the case studies considered in this work involve *in silico *data with known true parameters, a maximum iteration number is used as a termination criteria and the efficacy of each method is judged based on the accuracy of the respective estimates.

The SSA and DE algorithms were implemented using Message Passing Interface (MPI) in C++ and run on a Linux IBM computing cluster (CentOS; GNU C++ compiler (v4.1.1)). A combination of a long period random number generator [27] and multiple independent streams generator [28] were used to guarantee statistically independent streams of random numbers required for both the SSA and DE.

## Results

### Case Study 1: RNA dynamics in *E. coli*

**Figure 1:**
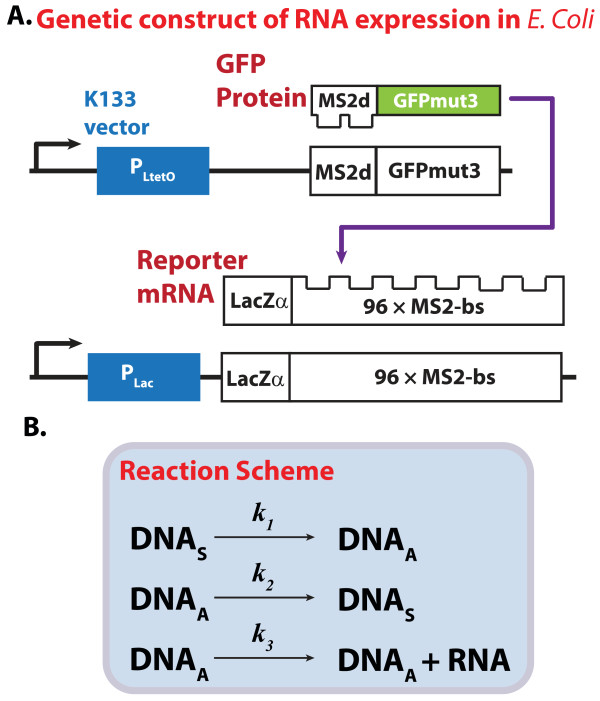
**mRNA Dynamics Model in Escherichia coli**. (A) The mRNA detection system comprises two genetic elements; a fluorescence protein fused with bacteriophage protein (MS2d) and a reporter mRNA containing tandem repeats of MS2-binding sites. The GFP binding site repeats facilitate imaging and quantification of cellular mRNA to single molecular level. (B) The transcriptional model constitutes 3 reactions with 3 rate constants. DNA_S _represents the silent form, while DNA_A _represents the activated form

The significance of intra-cellular noise arises from the low copy number of genetic materials and gene transcriptional machinery. Thus, the quantification of mRNA would experience a greater influence of such noise than that of proteins, which may have thousands of copies. A high resolution fluorescence microscopy method has been developed to quantify the molecular count of mRNAs in individual *Escherichia coli *cells [6]. This method is based on the amplification of MS2d-fused fluorescence protein signal by binding to a reporter RNA that has multiple MS2d receptor sites (Figure 1A). The transcriptional response was shown to rise and plateau after 70-80 minutes post induction [6]. The molecular counts of the transcripts were obtained by normalizing the fluorescence flux with that generated by a single tagged RNA molecule. A mass-action kinetic model of the average mRNA level was used to fit the experimental data to obtain the kinetic parameter values [6].

The first case study uses the CME model corresponding to the reactions and kinetic parameters proposed in the original work, as shown in Figure 1B and detailed in supplementary data [Additional File 1: Supplementary Table S1] [6]. Considering this model to be the true system, four experimental datasets of mRNA copy numbers with different replicates (*m *= 10, 20, 100, and 10,000) were simulated using the SSA. The simulated data were contaminated with measurement errors arising due to the normalization of the fluorescence flux, were taken to be discrete rounded values of normal random samples *N*(0,0.25), consistent with the actual wet-lab experiments [6]. The mRNA transcripts per cell generation were recorded every 0.5 minutes until 75 minutes, mimicking the original experimental protocol.

The parameter search was constrained to a space bounded by **k **∈ [0,5]. The density functions predicted by the CME were constructed using 10,000 SSA realizations with added i.i.d and *N*(0,0.25) noise. In the case of low replicate datasets (*m *= 10, 20, and 100), only the DFD-CDF method was applied, in which the CDF of the experimental data was constructed according to: [19]

(9) $$F_{e}\left(o_{l},t_{i}\right)=\frac{l-0.5}{m},$$

where *l *now denotes the index of the state in replicate vector after arranging the data in ascending order (i.e., *o*_1 _≤ *o*_2 _≤ *...*≤ *o*_*m*_). This construction implicitly uses the differences between sorted data values as the bin sizes. As stated earlier, since the DFD-PDF method requires the histograms of experimental data, which in the case of low replicate datasets, are highly inaccurate, this method was only performed for cell population data (*m *= 10,000). The DE optimization was implemented with a population size of 30 (10 × the number of parameters) for 4,000 generations and the optimization routine took about 1.5 hours for completion.

**Figure 2:**
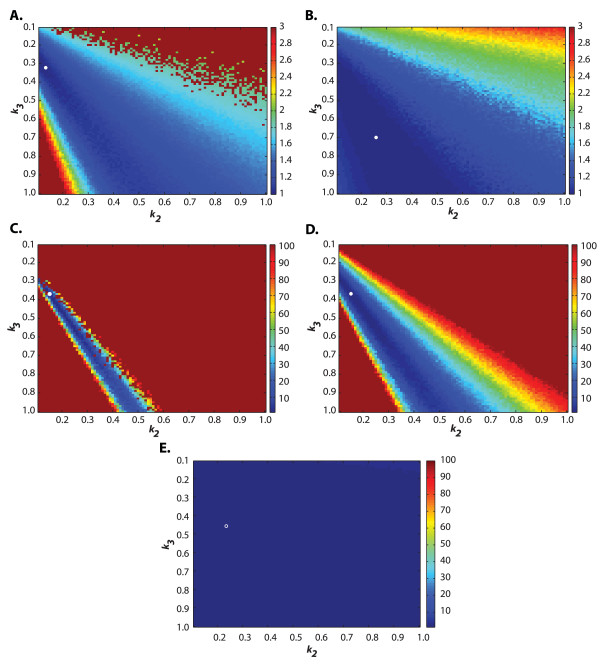
**Normalized objective function contours of the ML and DFD methods in the *E. coli *RNA dynamics model**. The parameter values *k*_2 _and *k*_3 _were varied between 0.1 and 1 while keeping the value of *k*_1 _at its original value. The normalization was done with respect to the optimal solution from each parameter estimation method, where the white circles represent the extrema on the normalized objective function plane. (A-B) Normalized objective function contours of the DFD-CDF and ML methods using sparse datasets (m = 10), respectively. (C-E) Normalized objective functions of the DFD-CDF, -PDF and ML methods using population datasets (m = 10,000).

Table 1 presents the parameter values estimated using the ML and DFD methods for all datasets. In general, the parameter estimates were closer to the true values with increasing number of replicates, as expected from the increase of information with higher replicates. The DFD(-CDF) method generally performed better than the ML. Amongst the parameters, *k*_1 _is the most accurately determined parameter by all methods. At higher replicates, the DFD-CDF method converged to the true solution faster than the PDF and ML methods, in this order, which could be attributed to the difference in the shape of the objective function surface. As seen in Figure 2A and 2C, the DFD-CDF criterion produced a higher surface curvature (second derivatives) than those of ML and DFD-PDF (Figure 2B, D and 2E). Using a larger population size and higher number of iterations (100 population members and 20,000 generations), the ML method was able to match the accuracy of the CDF estimates (see Table 1, *m = *10).

**Table 1:** Parameter estimation of RNA dynamics model in *E. coli*.

Replicates	ML			DFD-CDF			DFD-PDF		
	
	***k***_***1***_	***k***_***2***_	***k***_***3***_	***k***_***1***_	***k***_***2***_	***k***_***3***_	***k***_***1***_	***k***_***2***_	***k***_***3***_
10	0.0235 (0.0233)^a^	1.304 (0.3231)^a^	3.2201 (0.7232)^a^	0.02	0.1029	0.3643	-	-	-
20	0.0227	0.1095	0.2858	0.0371	0.2124	0.5263	-	-	-
100	0.0362	0.2930	0.5533	0.0273	0.1702	0.4121	-	-	-
10000	0.0279	0.2354	0.4872	0.0276	0.1659	0.4102	0.0273	0.1532	0.3837

Parameter estimates in the mRNA dynamics model in *E. coli*. The true parameter values are **k **= [0.0277; 0.1667; 0.4]. The search bound for the optimization algorithm was [0,5].

^a ^DE optimization performed with 100 population members and 20,000 generations

### Case Study 2: Galactose uptake model in *S. cerevisiae*

**Figure 3:**
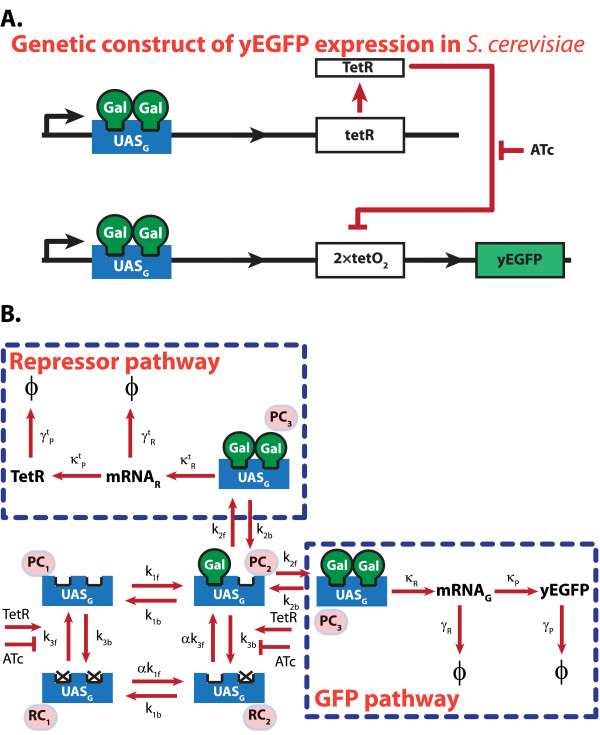
**Gene Expression Model for the Preferential Galactose Uptake in Yeast Cells**. (A) Genetic construct of the transcriptional control of the yeast-enhanced green florescent protein expression in the galactose utilization pathway of yeast. (B) The complete gene expression pathway includes (fast) reversible transformations among different promoter configurations and subsequent irreversible RNA and protein synthesis pathways. The reduced model assumes pseudo-equilibrium among the promoter configurations, and thus only describes dynamics of processes in the dashed boxes.

The inherent stochastic nature of gene expression can lead to diversified responses in a (clonal) cell population, even when subjected to uniform external conditions. This diversity has been demonstrated in a cell population using fluorescence techniques such as flow cytometery (FACS). The second case study used in this work looks at the problem of estimating CME parameters from a cell population data. The model describes an artificial genetic construct with the green fluorescence protein (GFP) gene downstream of a galactose activated promoter UAS_G _and a TetR repressor binding element 2xtetO_2 _(Figure 3A). In the presence of galactose, the GFP expression can be modulated rheostatically by varying the level of inducer ATc [29]. The original publication utilized a clonal population of *S. cerevisiae *(yeast) to investigate the inherent cellular noise in the GFP gene expression, which is measured as the heterogeneity of fluorescence among the cells.

The CME model adapted from this work captures the random transitions among all possible promoter states as shown in Figure 3B. The states PC_1_, PC_2 _and PC_3 _represent free/silent, intermediate complex, and pre-initiation complex promoter configurations, respectively, while the states RC_1 _and RC_2 _describe different forms of repressed promoter configurations. The transcriptional (RNA synthesis) and translational (protein synthesis) processes are modelled as single-step irreversible reactions (Figure 3B).

In the simplified model, the different promoter configurations are assumed to be in equilibrium, which reduces the model to a set of 8 irreversible reactions, 4 states, and 8 kinetic parameters, as shown in Figure 3B (dashed boxes) [29]. As in the first case study, this model was considered to be the true system and the molecular data of yEGFP and TetR were generated using SSA, giving 10^4 ^realizations at every 5 dimensionless time units up to 50 (or about 18 times the half life of yEGFP [30]). This condition corresponds to 440 minutes of post induction by 2% galactose and 40 ng ml^-1 ^ATc. To study the scalability of the proposed methods, the parameter estimation of the full network with 18 reactions, 9 states, and 15 kinetic parameters was also done using a second *in silico *dataset with 10^4 ^SSA realizations from the complete model. The details on the CME formulation for both the reduced and the complete model of the yEGFP gene expression pathway have been included in the supplementary data [Additional File 1: Supplementary Table S2 and S3].

Both ML and DFD methods were first applied to the reduced model, in which the DE optimization was done with 80 population members for 4000 generations, which took about 50 hours for convergence. The bounds on the parameter search space are given in Table 2. As mentioned above, the binning strategy in the DFD methods was based on the simulated experimental data, while the likelihood function in the ML method was constructed based on the histogram of SSA simulations. Table 2 presents the parameter estimates from the ML and the two DFD methods along with the true parameter values. As in the first example, the DFD-CDF method gave the most accurate estimates, followed by the DFD-PDF and ML methods, respectively. The parameter estimates from DFD-CDF gave yEGFP PDF that is in agreement with wet-lab data [Additional File 2]. As illustrated in Figure 2C, D &2E, the differences in the performance of these methods again arises from the steepness of the objective function plane. However, the lesser performing methods can potentially match the accuracy of the CDF method if population size and number of iterations in the DE optimization are increased.

**Table 2:** Parameter estimation of reduced yEGFP model in *S. cerevisiae*

Parameters	ML	DFD-CDF	DFD-PDF	Bounds	True values
*κ*_*R*_	1.1443	1	1.0478	[0,5]	1
*κ*_*P*_	1.0382	1.005	1.2174	[0,5]	1
*γ*_*R*_	4.5036	5.0306	5.7355	[0,10]	5
*γ*_*P*_	0.0128	0.0126	0.012	[0,5]	0.0125
$\kappa_{R}^{t}$	0.428	0.432	0.431	[0,5]	0.417
$\kappa_{P}^{t}$	2.1254	1.0542	1.24	[0,5]	1
$\gamma_{R}^{t}$	6.2433	2.9966	3.4982	[0,10]	3
$\gamma_{P}^{t}$	0.0102	0.0114	0.0115	[0,5]	0.0125

The scalability of the methods discussed in this work was evaluated by performing the estimation of the complete model. In this case, the DE optimization was performed using 150 population members for 4000 generations and took approximately 60 hours for convergence. In this case also, the CDF method again generally outperformed the PDF and ML (Table 3). But some of the parameters, especially those involving fast reversible processes, cannot be accurately identified from data. The lack of complete parameter identifiability is perhaps not surprising, when one considers that measurements of only few states are available and that the time scale of these measurements better reflects the slow kinetics of the irreversible processes.

**Table 3:** Parameter estimation of full yEGFP model in *S. cerevisiae*.

Parameters	Transcription processes				
	
	ML	DFD-CDF	DFD-PDF	Bounds	True value
*k*_1*f*_	0.4061	0.4082	0.4292	[0,5]	0.42
*k*_1*b*_	0.211	0.1171	0.8296	[0,5]	0.2485
*k*_2*f*_	74.1848	25.9882	99.7701	[0,100]	50
*k*_2*b*_	4.1423	18.8779	2.0815	[0,20]	10
*k*_3*f*_	3.2 × 10^-3^	3.87 × 10^-3^	0.0166	[0,5]	3.032 × 10^-3^
*k*_3*b*_	17.2405	19.9408	19.7665	[0,20]	10
*α*	0.1	0.0183	0.0211	[0,5]	0.025

**Irreversible processes**					

*κ*_*R*_	0.8939	0.9296	0.8078	[0,5]	1
*κ*_*P*_	2.0345	1.1103	1.0995	[0,5]	1
*γ*_*R*_	7.3543	5.2431	5.4116	[0,10]	5
*γ*_*P*_	0.0116	0.0124	0.012	[0,5]	0.0125
$\kappa_{R}^{t}$	0.4376	0.4157	0.4152	[0,5]	0.417
$\kappa_{P}^{t}$	1.7641	0.9755	1.3732	[0,5]	1
$\gamma_{R}^{t}$	4.3235	2.9034	3.9315	[0,10]	3
$\gamma_{P}^{t}$	0.0107	0.0116	0.0103	[0,5]	0.0125

Two other estimation criteria based on the maximum density function distance, in the form of

(10) $$k^{*}=\arg\underset{k}{\min}\sum_{i=1}^{m}\underset{L-1}{\max}\frac{\left|P_{e}\left(o_{l},t_{i}\right)-P\left(o_{l},t_{i}\right)\right|}{s_{l.i}}$$

and

(11) $$k^{*}=\arg\underset{k}{\min}\sum_{i=1}^{m}\underset{L-1}{\max}\frac{\left|F_{e}\left(o_{l},t_{i}\right)-F\left(o_{l},t_{i}\right)\right|}{S_{l,i}}$$

for PDF and CDF, respectively, have also been evaluated, showing similar performances and observations. The outcome of the application of these criteria to the estimation of parameters in the reduced and complete yEGFP gene expression pathway is described in supplementary data [Additional File 1: Supplementary Table S4 and S5].

### Case Study 3: Stochastic model of a synthetic toggle switch

**Figure 4:**
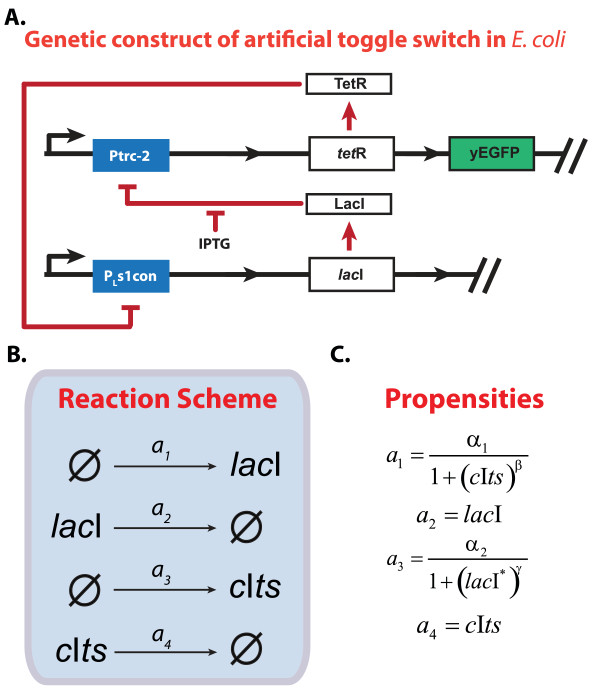
**Stochastic dynamics of synthetic gene toggle switch engineered in *E. coli***. (A) Synthetic circuit of the genetic toggle switch of *E.coli *[8]. (B) The genetic model of the toggle switch comprising of 4 reactions and (C) the corresponding propensity functions.

Multi-stability is often seen in biological networks, such as in λ-phage decision circuit [31], MAPK cascade [32], and cell cycle regulation [33]. In particular, bistability is a common motif encountered in cellular signalling pathways [34]. Motivated by this, a genetic toggle switch had previously been engineered in *E. coli *to show the ability to synthesize such motif. The synthetic switch consisted of two repressor-promoter pairs, with (i) *P*_*L*_*s1con-lac*I repressing *Ptrc*-2 promoter and (ii) vice versa *Ptrc*-2-*cIts *(thermal sensitive) repressing *P*_*L*_*s1con *promoter [8], such that they are mutually inhibitory (see Figure 4A). The switching behavior was visualized by means of green fluorescence protein (GFP), inserted downstream of *cIts*. The ON switch was accomplished by an inducer, isopropyl β-D-thiogalactosepyronoside (IPTG), that represses the activity of *lac*I (Figure 4A). By modulating the concentrations of the IPTG, the genetic toggle system could exhibit bistability with hysteresis [8].

A simple deterministic model was proposed to examine the behaviour of the toggle switch and to analyze different conditions of bistability [8]. The corresponding CME formulation is described in the Figure 4B and 4C[35]. Here, the propensity functions are taken directly from the deterministic model and they give effective rates of reaction following a canonical Hill equation. Taking this model to be the true system, *in silico *data of GFP fluorescence at IPTG concentration of 6 × 10^-5 ^M were simulated using 10^4 ^independent SSA realizations, emulating flow cytometry data.

As the ML performed consistently poorer than the DFD methods in the previous case studies, the stochastic rate constants here (*α*_1_, *α*_2_, *β*, *γ*, *η*, *K*) were estimated using the DFD-CDF and -PDF methods, with DE parameters: 150 population members and 4000 generations. Both CDF and PDF criteria took about 48 hours for completion. The parameter bounds and estimates are given in Table 4. Comparing to the true values, this case study, like the previous two, again showed that the DFD-CDF method performed better than DFD-PDF with more accurate and robust estimates of the kinetic rate constants. Performance of different estimation methods on another bistable system (Schlögl model) is presented in supplementary data [Additional File 1: Supplementary Table S6][Additional File 3].

**Table 4:** Parameter estimation of synthetic toggle switch in *E. coli*.

	DFD-CDF	DFD-PDF	Bounds	True value
*α*_1_	137.716	99.456	[0,200]	156.25
*α*_2_	15.644	15.391	[0,20]	15.6
*β*	2.309	2.543	[0,10]	2.5
*γ*	1.071	1.015	[0,10]	1
*η*	2.065	8.434	[0,10]	2.0015
*K*	7.331 × 10^-5^	5.831 × 10^-4^	[0,1]	6.0 × 10^-5^

## Discussion

In this work, three practical methods are proposed for the estimation of the parameters from (noisy) single cell datasets with low and high replicates. As the methods rely on a histogram construction of density functions from a finite sample of experimental data and Monte Carlo simulations, the objective function evaluation has a trade-off between low accuracy when using bins that are too wide, and high sensitivity to noise when bins are too small. In order to balance this trade-off, the binning was done such that the narrowest bin has at least ten occurrences. The noise associated with this binning strategy is also taken into account in the objective function in the DFD methods, which is modelled according to a binomial distribution.

The proposed methods are developed while considering a few practical issues when dealing with real biological datasets, such as data sparsity (low replicates), data noise and relatively coarse sampling intervals. The methods developed here do not require fast time-sampling like in [14], which might pose a restrictive constraint in practice. When population data are available, the DFD methods can fully exploit the additional information and rigorously handle the noise associated with the finite sample construction of a density function through the weighting factors. Although the examples considered in this work are represented by the CME, the methodologies developed in this work are generally applicable to parameter estimation of other stochastic models (e.g. Langevin), as long as the distribution density function can be constructed. Furthermore, the different methods developed in this work can be used to robustly estimate the rate constants of large scale gene expression networks as well as systems with multistability and general nonlinear propensity equations.

The case studies above showed that methods based on matching density function shapes between model and data generally performed better than maximizing likelihood function. Furthermore, the DFD-CDF distance is more sensitive to parameters than both the DFD-PDF and ML, and thus is the most effective method developed in this work. The higher sensitivity of the CDF with respect to parameter variations is expected as a result of the cumulative sum of the PDF sensitivity. This is evident from comparing the normalized objective function surfaces as shown in Figure. 2, in which the CDF objective functions have the steepest curvature. The increased curvature leads to a faster convergence to the minima in the DE optimization of the CDF than the PDF, though both methods eventually converge to optimal parameter estimates with similar accuracy. In addition, the CDF is generally less sensitive to noise from finite sampling as can be seen from the noise weighting factor *S*_*l,i *_when normalized with the respective probability, i.e. the coefficient of variation (CoV) $S_{l,i}/F_{e}\left(o_{l},t_{i}\right)=\sqrt{1-F_{e}\left(o_{l},t_{i}\right)}/n\sqrt{F_{e}\left(o_{l},t_{i}\right)}$. The monotonically decreasing CoV as a function *F*_*e*_(**o**_*l*_, *t*_*i*_) of indicates that the CDF construction becomes less affected by finite sampling noise with increasing *F*_*e*_(**o**_*l*_, *t*_*i*_).

Similar to the parameter estimation in deterministic models, parameter identifiability is a key issue in the estimation of the CME parameters. Such problem is commonly encountered in the parameter estimation of deterministic ODE models [36]. Following the same arguments from the deterministic estimation, the identifiability problem is caused by the limited information contained in the data about the parameters governing the fast transformations among the different promoter configurations. Such problem can be alleviated by getting additional measurements with a faster sampling rate and if possible, measuring the variables that are directly affected by the parameters, e.g. the fractions of promoters in each configuration of the second case study. An analogue of deterministic parameter identifiability analysis can be performed using the parametric sensitivity of the density function and experiments can be designed to maximize the degree of information in the data [35,37,38].

**Figure 5:**
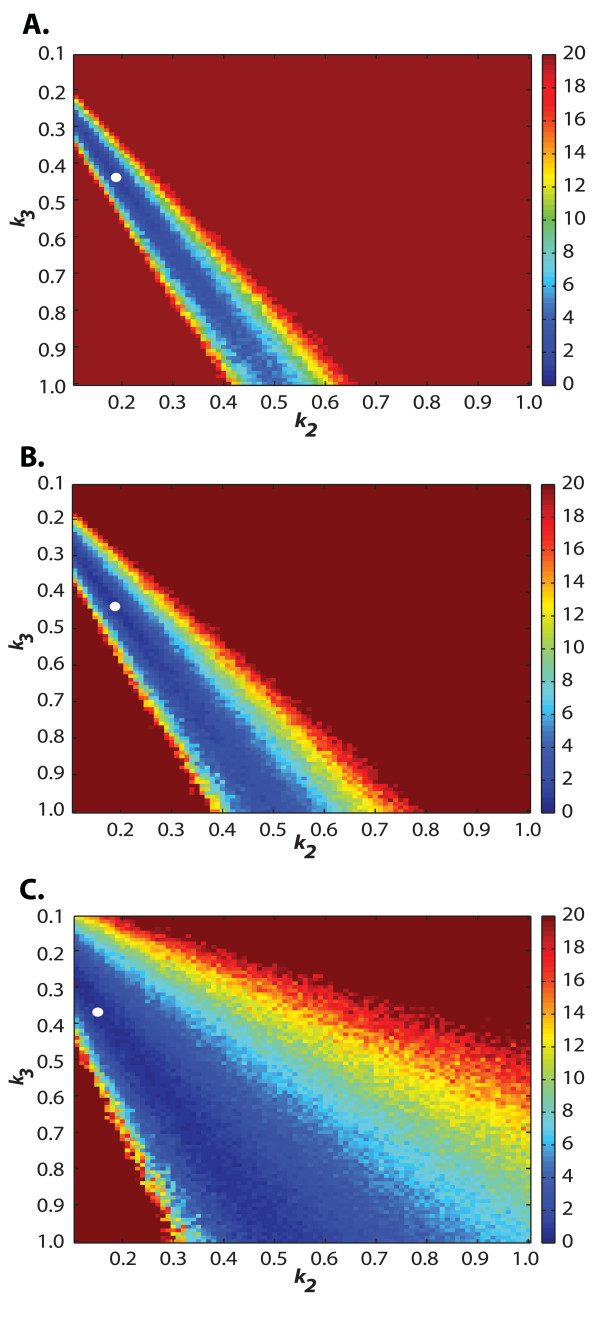
**Effect of the finite sampling noise on the parameter estimation of *E. coli *RNA dynamics model**. Normalized objective function contours of the DFD-PDF method for SSA realizations of 10,000 (A), 5000 (B), and 1000 (C). The parameter values *k*_*2 *_and *k*_*3 *_were varied between 0.1 and 1 while keeping the value of *k*_*1 *_at its original value. The normalization was done with respect to the optimal solution from each case, where the white circles represent the extrema on the normalized objective function plane.

Most of the computational cost of the parameter estimation related to CME is due to the large number of SSA realizations needed to construct the solution of the CME. Furthermore, every generation of DE requires multiple computations of the objective function according to the population size setting and each of population members in turn requires the SSA solution as mentioned previously. One way to alleviate the computational burden would be to lower the SSA realizations in constructing the density function. This would however increase the binning noise, and could possibly reduce the speed of convergence to the optimal solution and the accuracy of parameter estimates (see Figure 5A-C). Nevertheless, there is a diminishing return with increasing number of SSA realizations, since noise variance generally scales with the inverse of the number of samples (i.e. the standard deviation is only halved for every 4 times increase in the number of data). Alternatively, efficient approximation methods for simulating the CME can be used in place of the exact SSA [20,23,39-42], again at the cost of reduced estimation accuracy. In addition, the optimization parameters, namely population size and generations, can be further tuned for the proposed methods. Unfortunately, the relationship between these two parameters is most likely nonlinear and problem specific, which may require trial and error methods to find the best setting for a particular problem.

## Conclusions

The inherent stochasticity associated with low copy number processes in the cellular genetic milieu can introduce significant noise in gene expression profiles. The modelling of such noisy system requires a careful consideration of random processes and the parameters governing the probability of random events [1]. Three parameter estimation methods for stochastic models have been proposed based on the maximum likelihood criterion and density function distances of PDF and CDF. Since state density functions of stochastic systems are often constructed from a finite number of experimental data points or Monte Carlo realizations, a careful consideration has been taken to characterize the influence of noise arising from the histogram binning. Specifically, the effects of histogram noise are directly incorporated into the parameter estimation objective function as weighting functions. Applications to two case studies have shown that the proposed methods are both effective and practical. Amongst the proposed methods, the CDF-DFD method has been found to be the most efficient in estimating the kinetic rate constant than the others (i.e., the ML and DFD-PDF methods) due to the higher sensitivity of CDF to the parameters.

## Authors' contributions

SKP and RG conceived the project, SKP carried out all the simulations, performed the analyses and drafted the manuscript; RG provided project oversight and analyses, edited the manuscript. Both the authors read and approved the final manuscript.

## Supplementary Material

Additional file 1 **Supplementary tables of the manuscript file**. Six supplementary tables are included in this document; Table S1 describes the SSA formulation of the *E. coli *RNA dynamics model of the case study 1. Table S2 details the SSA formulation of the reduced yeast enhanced GFP galactose utilization pathway of the case study 2. Table S3 provides the SSA formulation of the complete gene expression model of the yEGFP galactose utilization pathway. Tables S5 and S6 give the parameter estimation results for the reduced and complete yEGFP gene expression models, respectively. The parameter estimation in these cases was done using the DFD methods involving the maximum distance measures (equation 10 and 11 in the main text). Table S6 lists the parameter estimation results of the Schlögl model. Click here for file

Additional file 2 **Supplementary figure of the manuscript file**. Comparison of actual experimental data and CME model prediction using SSA simulations with the parameters estimated in case study 2. Click here for file

Additional file 3 **Supplementary text of the manuscript file**. Details of the SSA formulation and the parameter estimation method used in the Schlögl case study. Click here for file
