# Supplementary material for: Global parameter estimation methods for stochastic biochemical systems
Source: BMC Bioinformatics. 2010 Aug 6;11:414. doi: 10.1186/1471-2105-11-414 (PMC2928803; doi:10.1186/1471-2105-11-414)
Supplement: Additional file 1 — Supplementary tables of the manuscript file. Six supplementary tables are included in this document; Table S1 describes the SSA formulation of the E. coli RNA dynamics model of the case study 1. Table S2 details the SSA formulation of the reduced yeast enhanced GFP galactose utilization pathway of the case study 2. Table S3 provides the SSA formulation of the complete gene expression model of the yEGFP galactose utilization pathway. Tables S5 and S6 give the parameter estimation results for the reduced and complete yEGFP gene expression models, respectively. The parameter estimation in these cases was done using the DFD methods involving the maximum distance measures (equation 10 and 11 in the main text). Table S6 lists the parameter estimation results of the Schlögl model. [file 1471-2105-11-414-S1.PDF]

Table S1: SSA formulation of the RNA dynamics model.

Reactions	SSA implementation<br>Propensities	Stoichiometric change
1	$k_1 \times DNA_S$	$DNA_S = 0; DNA_A = 1$
2	$k_2 \times DNA_A$	$DNA_S = 1; DNA_A = 0$
3	$k_3 \times DNA_A$	$mRNA = mRNA + 1$

Table S2: SSA formulation for the reduced model of the reduced - yEGFP galactose utilization pathway.

Reactions	SSA implementation<br>Propensities	Stoichiometric change
1	$\kappa_R \langle PC_3 \rangle$	$mRNA_G = mRNA_G + 1$
2	$\gamma_R \times \mathrm{mRNA_G}$	$mRNA_G = mRNA_G - 1$
3	$\kappa_P \times \mathrm{mRNA_G}$	yEGFP = yEGFP + 1
4	$\gamma_P \times \mathrm{yEGFP}$	yEGFP = yEGFP - 1
5	κ_R^t	$mRNA_R = mRNA_R + 1$
6	$\gamma_R^t \times \mathrm{mRNA_R}$	$mRNA_R = mRNA_R - 1$
7	$\kappa_P^t \times \mathrm{mRNA_R}$	TetR = TetR + 1
8	$\gamma_R^t \times \mathrm{TetR}$	TetR = TetR - 1

Table S3: SSA formulation for the complete model of the full yEGFP galactose utilization pathway.

Reactions	SSA implementation<br>Propensities	Stoichiometric change
Transcription reversible process		
1	$k_{1f} \times \mathrm{PC_1}$	$\mathrm{PC_1} = 0;\ \mathrm{PC_2} = 1$
2	$k_{1b} \times \mathrm{PC_2}$	$\mathrm{PC_1} = 1;\ \mathrm{PC_2} = 0$
3	$k_{2f} \times \mathrm{PC_2}$	$\mathrm{PC_2} = 0;\ \mathrm{PC_3} = 1$
4	$k_{2b} \times \mathrm{PC_3}$	$\mathrm{PC_2} = 1;\ \mathrm{PC_3} = 0$
5	$k_{3f} \times \mathrm{PC_1}$	$\mathrm{PC_1} = 0;\ \mathrm{RC_1} = 1$

6	$k_{3b} \times \mathrm{RC}_1$	$\mathrm{PC}_1 = 1;\ \mathrm{RC}_1 = 0$
7	$\alpha k_{1f} \times \mathrm{RC}_1$	$\mathrm{RC}_1 = 0;\ \mathrm{RC}_2 = 1$
8	$k_{1b} \times \mathrm{RC}_2$	$\mathrm{RC}_1 = 1;\ \mathrm{RC}_2 = 0$
9	$\alpha k_{3f} \times \mathrm{PC}_2$	$\mathrm{PC}_2 = 0;\ \mathrm{RC}_2 = 1$
10	$k_{3b} \times \mathrm{RC}_2$	$\mathrm{PC}_2 = 1;\ \mathrm{RC}_2 = 0$
	Irreversible process	
1	$\kappa_R \langle PC_3 \rangle$	$mRNA_G = mRNA_G + 1$
2	$\gamma_R \times \mathrm{mRNA_G}$	$mRNA_G = mRNA_G - 1$
3	$\kappa_P \times \mathrm{mRNA_G}$	yEGFP = yEGFP + 1
4	$\gamma_P \times \mathrm{yEGFP}$	yEGFP = yEGFP - 1
5	κ_R^t	$mRNA_R = mRNA_R + 1$
6	$\gamma_R^t \times \mathrm{mRNA_R}$	$mRNA_R = mRNA_R - 1$
7	$\kappa_P^t \times \mathrm{mRNA_R}$	TetR = TetR + 1
8	$\gamma_R^t \times \mathrm{TetR}$	TetR = TetR - 1

Table S4: Estimated kinetic rate constants of the reduced yEGFP genetic transcriptional process (see Fig. 3 (dashed boxes) in main text) using the DFD method with maximum distance measure.

Parameters	CDF	PDF	Bounds	True values
κ_R	1.0196	1.0311	$\in [0,5]$	**1**
κ_P	0.9997	1.0735	$\in [0,5]$	**1**
γ_R	4.92	5.1962	$\in [0,10]$	**5**
γ_P	0.0127	0.0127	$\in [0,5]$	**0.0125**
κ_R^t	0.4373	0.4285	$\in [0,5]$	**0.417**
κ_P^t	1.1713	1.1122	$\in [0,5]$	**1**
γ_R^t	3.276	3.1543	$\in [0,10]$	**3**
γ_P^t	0.0115	0.01103	$\in [0,5]$	**0.0125**

Table S5: Estimated kinetic rate constants of the complete yEGFP genetic transcriptional process (see Fig. 3 in main text) using the DFD method with maximum distance measure.

**Parameters**	**Transcription processes**				**Parameters**	**Irreversible processes**			
	**CDF**	**PDF**	**Bounds**	**True values**		**CDF**	**PDF**	**Bounds**	**True values**
k_{1f}	0.4428	0.4192	$\in [0,5]$	**0.42**	κ_R	0.9296	1.1078	$\in [0,5]$	**1**
k_{1b}	0.1468	1.0296	$\in [0,5]$	**0.2485**	κ_P	1.1451	2.2095	$\in [0,5]$	**1**
k_{2f}	98.2117	45.7701	$\in [0,100]$	**50**	γ_R	5.6745	6.4432	$\in [0,10]$	**5**
k_{2b}	19.9595	1.0834	$\in [0,20]$	**10**	γ_P	0.0106	0.0222	$\in [0,5]$	**0.0125**
k_{3f}	9.4113×10^{-3}	0.0232	$\in [0,5]$	**3.032×10^{-3}**	κ_R^t	0.4062	0.5323	$\in [0,5]$	**0.417**
k_{3b}	15.042	12.3425	$\in [0,20]$	**10**	κ_P^t	1.1087	2.3425	$\in [0,5]$	**1**
α	0.0707	0.0834	$\in [0,5]$	**0.025**	γ_R^t	3.4129	4.1232	$\in [0,10]$	**3**
					γ_P^t	0.013	0.0324	$\in [0,5]$	**0.0125**

Table S6 - Parameter estimation of Schlögl model.

**Paremeters**	**DFD-CDF**	**DFD-PDF**	**Bounds**	**True values**
$k_1[A]$	0.4563	0.3125	[0,1]	0.5
k_2	3.04418	3.0123	[0,5]	3.0
k_3	0.9148	1.1362	[0,5]	1.0
k_4	1.1083	1.4563	[0,5]	1.0
