# Supplementary material for: Global parameter estimation methods for stochastic biochemical systems
Source: BMC Bioinformatics. 2010 Aug 6;11:414. doi: 10.1186/1471-2105-11-414 (PMC2928803; doi:10.1186/1471-2105-11-414)
Supplement: Additional file 2 — Supplementary figure of the manuscript file. Comparison of actual experimental data and CME model prediction using SSA simulations with the parameters estimated in case study 2. [file 1471-2105-11-414-S2.PDF]

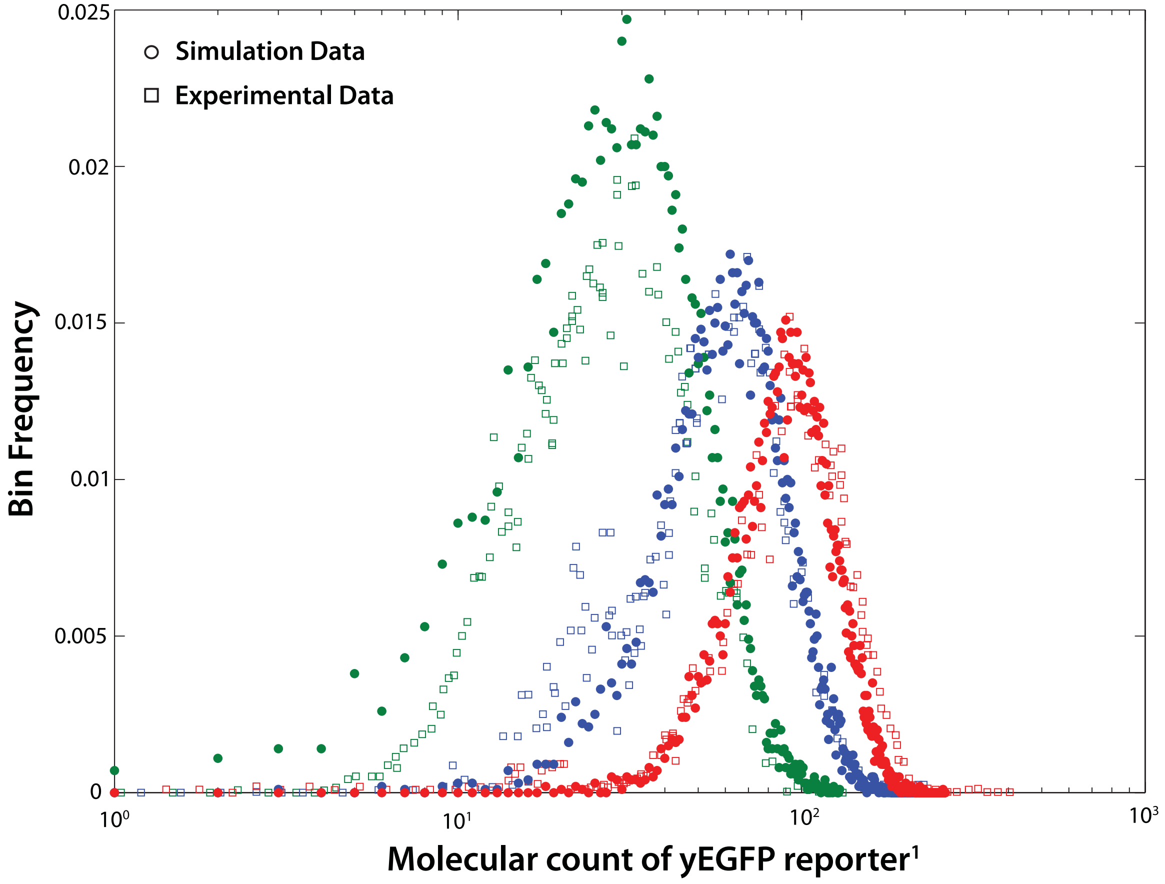


[1]Blake WJ, M KA, Cantor CR, Collins JJ: **Noise in eukaryotic gene expression.** *Nature* 2003, **422:** 633- 637

**Supplementary Figure 1: Comparison of actual experimental data and CME model prediction using SSA and parameters estimated in case study 2 (galactose uptake model in *S. cerevisieae*) (green – 150 minutes, blue – 290 minutes, and red – 440 minutes post-induction of 40 ng/ml ATc) [1].**

1. Blake WJ, M KA, Cantor CR, Collins JJ: **Noise in eukaryotic gene expression.** *Nature* 2003, **422:**633-637.
