# Supplementary material for: Global parameter estimation methods for stochastic biochemical systems
Source: BMC Bioinformatics. 2010 Aug 6;11:414. doi: 10.1186/1471-2105-11-414 (PMC2928803; doi:10.1186/1471-2105-11-414)
Supplement: Additional file 3 — Supplementary text of the manuscript file. Details of the SSA formulation and the parameter estimation method used in the Schlögl case study. [file 1471-2105-11-414-S3.PDF]

Supplementary Text

Case Study 4: The Schlögl Model

The Schlögl reaction has commonly been used as an example of a bistable system [1-3]:

$$\begin{aligned} &A \underset{k_2}{\overset{k_1}{\rightleftharpoons}} X \\ &3X \underset{k_4}{\overset{k_3}{\rightleftharpoons}} 2X + B \end{aligned} \qquad [1]$$

The concentrations of A and B are typically assumed constant (buffered). The propensity functions for the above reaction can be formulated as:

$$\begin{aligned} a_1 &= k_1 \left[A\right] \Omega \\ a_2 &= k_2 X \\ a_3 &= k_3 X \frac{X-1}{\Omega} \frac{X-2}{\Omega} \\ a_4 &= k_4 \left[B\right] X \frac{X-1}{\Omega} \end{aligned} \qquad [2]$$

where Ω (Ω = 10 units is chosen here) is called the extensivity, a parameter that is proportional to the system volume. The actual values of the kinetic rate constants used for the SSA realizations are presented in the supplementary table S6.

The molecular data of component X is formed as a density function constructed using 10^4 independent realizations of SSA, simulated for a time period of 20 units and recorded for every 4 time units. Parameter estimation was performed using the DFD method with both CDF and PDF criteria. The DE optimization was implemented with a population size of 40 and for 4000 generations and the estimation took about 72 hours for completion. Supplementary table S6 shows the results of the parameters

estimation, which again indicates a better performance of the DFD-CDF method compared to the DFD-PDF method.

References

1. Gaspard P (2004) Fluctuation theorem for nonequilibrium reactions. J Chem Phys 120: 8898-8905.
2. Schlögl F (1971) On Thermodynamics Near a Steady State. Z Physik 248: 446-458.
3. Schlögl F (1972) Chemical Reaction Models for Non-Equilibrium Phase Transitions. Z Physik 253: 147-161.
